# A Comparison between Predictions of the Miller–Macosko Theory, Estimates from Molecular Dynamics Simulations, and Long-Standing Experimental Data of the Shear Modulus of End-Linked Polymer Networks

**DOI:** 10.1021/acs.macromol.3c02544

**Published:** 2024-04-17

**Authors:** Ioanna Ch. Tsimouri, Fabian Schwarz, Tim Bernhard, Andrei A. Gusev

**Affiliations:** Department of Materials, ETH Zürich, CH-8093 Zürich, Switzerland

## Abstract

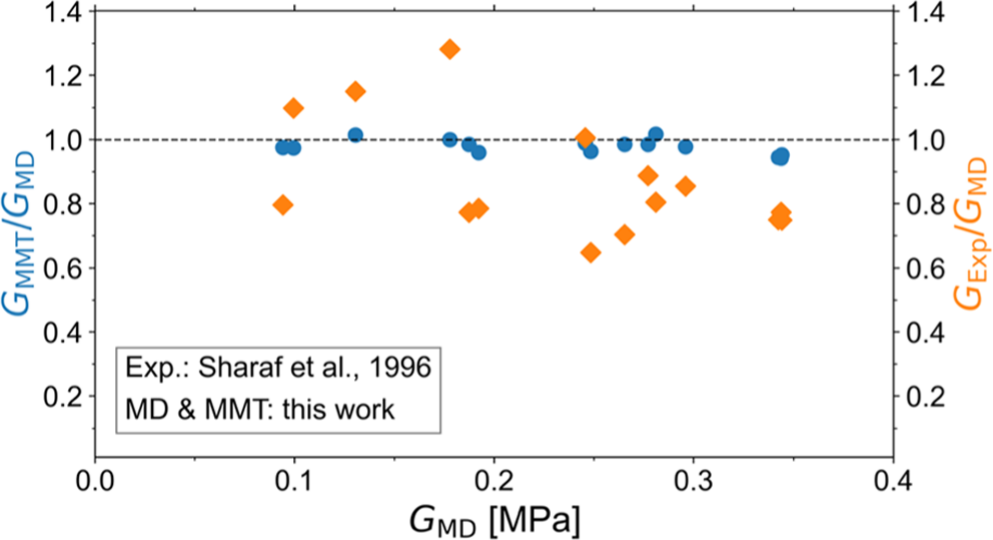

Long-standing experimental data on the elastic modulus of end-linked poly(dimethylsiloxane) (PDMS) networks are employed to corroborate the validity of the Miller–Macosko theory (MMT). The validity of MMT is also confirmed by molecular dynamics (MD) simulations that mimic the experimentally realized networks. It becomes apparent that for a network formed from bulk, where the fractions of the loops are small, it is sufficient to account for the topological details of a reference tree-like network, i.e., for its degree of completion, junction functionalities, and trapped entanglements, in order to practically predict the modulus. However, a mismatch is identified between the MMT and MD simulations in relating the fraction of the soluble material to the extent of reaction. A large contribution of entanglements to the modulus of PDMS networks prepared with short precursor chains is presented, suggesting that the elastic modulus of commonly used end-linked PDMS networks is in fact entanglement-dominated.

## Introduction

Rubber-like materials, or in more modern terms, elastomers, have attracted the attention of the scientific community for almost a century because of their increasing importance and their vast range of industrial applications. Adhesives and coating, tires, shock isolators, vibration and noise absorbers, corrosion protectors, electrical and thermal insulators, seals, cardiac valve replacements, and lenses are some of the most common applications of elastomers,^1−3^ while their market value exceeded 80 billion U.S. dollars in 2019.^4^ The most distinguishing and essential characteristic of elastomeric materials is their large elastic deformability, which enables them to significantly deform under relatively small stresses and recover their initial shape upon the removal of the stresses.^5,6^ This distinct behavior of elastomers stems from the topology of their molecular structure. A profound understanding of the molecular topology of elastomers and the relation to their mechanical properties is, therefore, of paramount importance.

Elastomers, which are amorphous polymers above their glass transition temperature, owe their unique mechanical properties to the nature of the chemically linked polymer chains that constitute their network structure. The elastic deformability of rubber-like materials has been generally conceptualized through the conformational rearrangements that take place within the polymer network, which decrease its configurational entropy during stretching.^5^ This entropy change is what drives a polymer network back to its original undeformed state. Based on the configurational statistics of long-chain molecules, Kuhn^7^ was the first who successfully formulated a theory for rubber elasticity. The theory of Kuhn was later finely developed by Wall,^8−10^ and Flory and Rehner,^11^ establishing the statistical molecular theory of rubber elasticity. The theory, typically termed the affine network model (ANM), assumed ideal phantom chains, i.e., chains that can pass through one another, whose mean square end-to-end distance obeys Gaussian statistics, and cross-linkers, i.e., molecules that bond the chains together covalently, whose displacements are affine in the macroscopic deformation as if they are fixed on the elastic background of the network.^5,12^ However, the agreement between theoretical predictions of the modulus and experiments at that stage was unsatisfactory, primarily from ignoring entanglements.^5,6,12^

Few years after the development of ANM, toward the end of the 1940s, the theory of James and Guth,^13^ who endeavored to more realistically describe the elasticity of polymer networks on the molecular basis by allowing the cross-linkers to fluctuate around their mean positions, marked a milestone in the development of rubber elasticity.^12^ Through this relaxation in the movement of the junctions, the equilibrium shear modulus of an ideal network acquired the same form as ANM and differed only by the front factor that includes the number density of the network cross-linkers (see Appendix).^14^ This model is generally known as the phantom network model (PNM), even though both ANM and PNM assume networks with phantom chains.

To account for network imperfections, James and Guth^13^ further elaborated their theory by considering a progressive instead of an instantaneous cross-linking and derived an expression for the absolute value of the equilibrium shear modulus. The expression for the modulus is given again by the same form as ANM and PNM, and it is different only by the front factor that includes Γ, the topological factor that describes the size of the chains in the undeformed state (see Appendix). Unfortunately, the relationship for the topological factor depends on the history of network formation and is experimentally unattainable.^5^ However, the affine network theory (ANT), as it is termed, has been shown to be accurate in describing the elasticity of networks that allow their chains to cross each other and themselves (i.e., networks composed of phantom chains) by numerical simulations that are able to access all of the information required to compute Γ.^15−19^ Almost two decades followed along with several chemistry attempts in preparing rubber-like networks by random cross-linking and a great deal of effort in relating the equilibrium shear modulus with the degree of cross-linking, which however did not come to fruition since none of the existing statistical theories at the time could accurately describe the properties of rubbers on molecular grounds.^5^

Around 1970, Langley^20,21^ sparked an interest in the additional contribution of the trapped entanglements to the elastic modulus. During this time, the pioneering work of De Gennes on the reptation of polymer chains in melts^22^ and Edwards’s tube model,^23,24^ which provided the first statistical mechanics description of entanglements, completely transformed scientists’ perception of entanglements that were mostly ignored before. Later, Graessley and co-workers,^25−27^ following the idea of Langley, provided a simple empirical methodology to predict the shear modulus of rubber-like materials at the limit of small deformations. In particular, they assumed that there are two contributions to the modulus: one stems from the Gaussian phantom description and one stems from the trapped entanglements. The latter is a product of two terms, the trapping factor, *T*_e_, that was proposed by Langley and depends on topological features, and the plateau modulus of a melt of high-molar-mass linear chains of the corresponding polymer, *G*_N_^0^, that depends on the chemical constitution.^28,29^

At that time, the concepts of elastically effective strands and junctions were introduced.^28^ Elastically effective strands are strands that are able to deform and store energy when the network undergoes deformation.^14^ Based on the classical theory of Flory and Stockmayer that describes percolation processes, Miller and Macosko derived a theory (MMT) to obtain average properties of nonlinear polymers and provided useful expressions for calculating network structural parameters of imperfect networks that inherently discount ineffective chains that do not contribute to elasticity.^30,31^ Through MMT, the structural parameters of real networks can be estimated utilizing the information on their soluble material fraction, *W*_sol_, which is the weight (mass) fraction of strands and cross-links that can be removed by swelling the sample and measured by straightforward extraction experiments.^32^ This provides an implicit way of measuring the extent of polymerization (cross-linking). It can also be measured explicitly by high-resolution ^1^H magic-angle spinning (MAS) NMR spectroscopy^33^ and Fourier transform infrared (FT-IR) spectroscopy,^34^ which however are sometimes impractical and of moderate sensitivity and, therefore, they are not always reliable to directly measure the extent of reaction. By combining their expression for the concentration of the effective network chains with Langley’s and Graessley’s suggestion for the entanglements contribution, Miller and Macosko provided a formula to predict the equilibrium modulus of rubber-like materials^30^
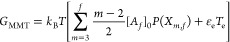
1where *T*_e_ represents the trapping factor [eq 14 in Appendix], *P*(*X*_*m,f*_) is the probability that a cross-linker *A*_*f*_ of functionality *f* is an elastically effective cross-linker of degree *m* [eq 15], and [A_*f*_]_0_ is the initial molar density of the cross-linkers before the cross-linking onset [eq 17]. In eq 1, ε_e_ is an effective concentration of pseudo-tetrafunctional junctions. The term ε_e_*k*_B_*T* ≡ *G*_e_(1) represents the entanglement modulus of a fully polymerized stoichiometric network (*T*_e_ = 1), which has been connected to the plateau modulus of a high-molar-mass linear polymer melt, *G*_N_^0^; the value of *G*_e_(1) is expected to be between *G*_N_^0^ and 5G_N_^0^/4, with the Doi–Edwards factor 5/4 related to contraction of melt chains into their tube after deformation.^23^ For a fully polymerized network prepared in the unstrained state, this contraction is prevented by the cross-links and hence, an increased value of *G*_e_(1) as compared to the measured melt plateau modulus *G*_N_^0^.

Since the 1960s and 1970s, when the science of polymer chemistry flourished and new synthetic and characterization techniques were developed, end-linked polymer networks, commonly designated as model networks, have been employed in validating the theories of rubber elasticity, owing to the known structural information they offer. As opposed to their uncontrolled randomly cross-linked counterparts, cross-linking polymer chains solely by their ends results in networks with cross-linkers of known functionality and with chains of known molecular weight and molecular weight distribution between cross-linkers.^35^ Before that time, only insufficient quantitative information was available to correlate the structural properties of elastomeric networks with their mechanical properties, even though the classical theories of rubber elasticity were derived already a few decades earlier.^3,36^ Yet, even after the intensive studies of model rubber-like networks, the conclusions regarding the validity of classical theories were conflicting.^37−43^ The origin behind this confusion was in the apparent discrepancy between the theoretical predictions and experimental measurements at the limits of small deformations, with its source being the contribution of interchain entanglements to the small-deformation equilibrium modulus, even though the results of Sharaf, Mark, and Alshamsi^43^ indicated that Langley’s idea was not inconsistent with their data. At present, after years of debate, development of meticulous characterization techniques, and significant increase in computer power, the significance of entanglements in the elasticity of polymer networks has been proven beyond any doubt by both experiments and simulations.^14,16,19^

Recently, molecular dynamics simulations shed light upon the validity of the Miller–Macosko theory (MMT) in describing the elastic modulus of model networks and confirmed the additivity of the phantom and entanglement contributions.^16^ Gusev and Schwarz^16^ studied stoichiometric (*r =* 1, with *r* being the ratio of the cross-linkers active groups to the precursor polymer active groups in the initial mixture) tri- and tetrafunctional end-linked polymer networks, and showed that ε_e_ does not depend on the molecular weight of the network strands. Their work also demonstrated that one can use PNM and simply add the measured melt plateau modulus *G*_N_^0^ to predict the elastic modulus of real well-developed networks (with an extent of reaction, *p*, close to unity). However, they found that for more realistic extents of reaction, even though the addition assumption is still accurate, PNM cannot accurately predict the modulus due to the presence of dangling and free structures. In this case, the nonlinear MMT, eq 1, provided accurate estimates with ε_e_*k*_B_*T = G*_e_(1) being a fitting parameter, whose value, however, is expected to be between the measured melt plateau modulus *G*_N_^0^ and 5*G*_N_^0^/4; for PDMS, 0.20 MPa ≤ *G*_e_(1) ≤ 0.25 MPa.

Here, to assess the accuracy of MMT for nonstoichiometric networks, we revisit some long-standing experimental data on the elastic modulus of end-linked PDMS polymer networks prepared by Sharaf et al.^43^ in the 1990s using nearly monodispersed PDMS precursor chains of various molecular weights. This experimental study^43^ was selected for two reasons. First, the carefully prepared tetrafunctional PDMS networks were well characterized, and the precursor chains were practically monodispersed, which also makes them well suitable for computational replication. Moreover, the *W*_sol_ was measured, and the authors studied various combinations of molecular weights of the precursor chains, *M*_n_, and values of *r*. The second reason is that the authors concluded that there is no significant contribution from trapped entanglements to the small-strain modulus. It would be, therefore, tempting to show that this argument is simply not valid.

In this work, our intention is to provide strong evidence for the validity of the MMT expression [eq 1] as well as to corroborate the consequent substantial contribution of trapped entanglements to the shear modulus of rubber-like materials. For this, we revisit the experimental data of Sharaf et al.^43^ and compare them with both MMT predictions and MD estimates that incorporate the topological information on the realized networks.

## Theory and Simulation Section

### MMT Predictions

To obtain predictions of the Miller–Macosko nonlinear polymerization theory, we used the values of *r* and *W*_sol_ provided by Sharaf et al.^43^ in their Table 1 and calculated the extent of reaction *p* using
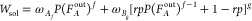
2with ω_*A*_*f*__ and ω_*B*_*g*__ being the mass fractions of the cross-linkers with functionality *f*, *A*_*f*_, and the precursor chains with functionality *g*, *B*_*g*_, in the mixture, respectively, from which eq 1 can be readily used with ε_e_*k*_B_*T* = *G*_e_(1) being a free fitting parameter. *P*(*F*_A_^out^) represents the probability of the event that looking out from a reactive site of a network junction chosen at random leads to a finite chain rather than to the infinite network (see Appendix). For the studied networks, *g* = 2 and *f* = 4 and *P*(*F*_A_^out^) is given by *P*(*F*_A_^out^) = [1/(*rp*^2^) – 3/4]^1/2^ – 1/2.

**Figure 1:**
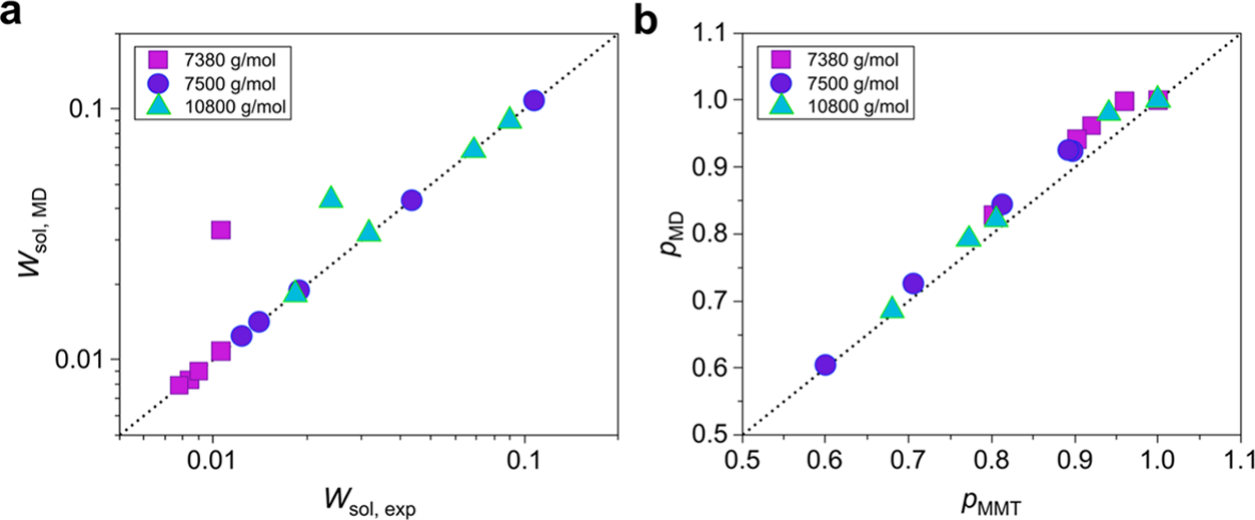
(a) Measured and MD results for the weight fraction of the soluble material *W*_sol_. (b) Estimated extent of reaction from MD and calculated extent of reaction from MMT using eq 2; the experimental *W*_sol_ values are used as input in both methods. The dotted lines are *y* = *x* and serve as a guide to the eye. Using the pairs of *p*_MMT_ and *p*_MD_ values from (b) as input for eq 3, the ratio of the corresponding moduli is 0.85 ± 0.04.

The experimentally measured shear modulus of each network was obtained by elongation tests with extracted samples with the soluble material being removed before testing. To account for this, we used the following scaling
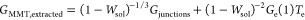
3
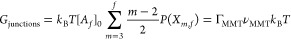
4where *G*_MMT,extracted_ represents the predicted MMT modulus in the unswollen state and [*A*_*f*_]_0_ = *rρN*_*A*_/(2*M*_n_), with ρ being the density of PDMS and the functionalities being replaced by *g* = 2 and *f* = 4. The scaling of the junctions contribution to elasticity, *G*_junctions_, is known from the effect of swelling on the mechanical properties of cross-linked networks based on statistical mechanics foundations.^5^ However, there is an ambiguity regarding the scaling of the contribution of entanglement to elasticity. Here, we provide a simple approach to assess the effect of deswelling on the entanglement term. Let us assume that the probability, , that a randomly chosen strand of a network with *p* < 1 is elastically effective, i.e., that looking out from each end of the strand leads to an infinite network, is given by
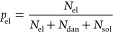
5in the swollen state (before extraction of the soluble material) and by
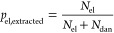
6in the unswollen state (after extraction of the soluble material), where *N* represents the number of chains with the subscripts el, dan, and sol corresponding to elastic, dangling, and soluble, respectively. It can be then shown that
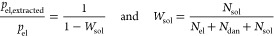
7which results in the scaling (1 – *W*_sol_)^−2^*G*_e_(1)*T*_e_, since *G*_e_(1) is independent of *W*_sol_, cf. eq 3. The scaling of eqs 3–7 was confirmed by comparing direct MD stress relaxation predictions of the shear modulus of a few networks with and without the soluble fraction. And it was also tested by comparing MMT predictions of eq 3 for the modulus of all studied microstructures with and without the soluble fraction, with a resulting mean ratio of the moduli of 0.96 ± 0.03. As seen from Figure 1a and also Table 1, the *W*_sol_ values are typically between 1 and 10%, corresponding to a linear rescaling of ca. 0.3–3%.

**Table 1:** Summary of Experimental Systems and Computer MD Models^a^

experimental				MD					
*M*_n_ [g/mol]	*r*	*W*_sol_	*G* [MPa]	bonds	*p*	*w*_dang_	*w*_1_	*w*_2_	*G* [MPa]
7408	0.867	0.0328	0.220	45	1.000	0.2143	0.0286	0.0110	0.2456
7408	0.947	0.0083	0.217	45	0.998	0.0962	0.0317	0.0132	0.3437
7408	0.987	0.0090	0.223	45	0.962	0.0907	0.0310	0.0145	0.3423
7408	1.015	0.0079	0.218	45	0.942	0.0852	0.0302	0.0135	0.3442
7408	1.142	0.0108	0.187	45	0.828	0.1444	0.0263	0.0111	0.2809
7569	0.990	0.0141	0.246	46	0.924	0.1641	0.0306	0.0128	0.2771
7569	1.004	0.0124	0.253	46	0.925	0.1385	0.0304	0.0130	0.2958
7569	1.090	0.0189	0.187	46	0.845	0.1809	0.0280	0.0119	0.2654
7569	1.226	0.0431	0.145	46	0.726	0.2892	0.0254	0.0080	0.1874
7569	1.420	0.1076	0.075	46	0.605	0.4299	0.0210	0.0055	0.0941
10 790	0.836	0.0434	0.151	66	1.000	0.2567	0.0263	0.0083	0.1922
10 790	0.923	0.0182	0.161	66	0.980	0.1653	0.0274	0.0115	0.2483
10 790	1.036	0.0683	0.150	66	0.792	0.3551	0.0197	0.0061	0.1304
10 790	1.060	0.0319	0.228	66	0.821	0.2797	0.0230	0.0074	0.1778
10 790	1.200	0.0898	0.109	66	0.686	0.4239	0.0173	0.0060	0.0993

a For the MD models, the weight fractions *w* are given before the extraction of the soluble fraction, whereas both experimental and MD shear moduli are for extracted network systems. The weight fractions of primary and secondary loops are labeled as *w*_1_ and *w*_2_, respectively.

### Molecular Dynamics Simulations

To carry out the Molecular Dynamics (MD) simulations, a modified Kuhn-scale PDMS Kremer–Grest (KG) model is used.^44^ In this model, the polymer chains are coarse-grained and are represented as chains of beads. All beads interact with each other by the Weeks–Chandler–Andersen potential:
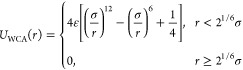
8

Here, ε = *k*_B_*T* is the Lennard-Jones (LJ) unit of energy, σ is the LJ unit of length, and the cutoff is 2^1/6^σ = 0.691 nm. Furthermore, beads that are bonded to one another interact via a finite extensible nonlinear elastic (FENE) potential:
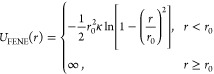
9

Here, *r*_0_ = 1.5σ = 0.924 nm, κ = 30ε/σ^2^ = 0.325 J/m^2^, as well as a bead density of 0.85σ^–3^ = 3.636 nm^–3^ are used according to refs (45,46). Finally, the modified KG model contains a bond angle term:^47,48^
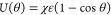
10where χ = 0.013.^44^ The mapping from the LJ units to SI units was achieved by comparing the results of melt simulations with experimental data.^44^ For PDMS at a reference temperature of *T* = 298 K, this resulted in 1ε = 4.11 × 10^–21^ J, 1σ = 0.616 nm, and 1*m* = 2.67 × 10^–25^ kg, which corresponded to a bead molar mass of around 161 g/mol.

The MD simulations are carried out using the Large-Scale Atomic/Molecular Massively Parallel Simulator (LAMMPS)^49^ on the ETH Zürich Euler cluster.^50^ The simulations are performed in the *NVT* ensemble (constant number of particles, constant volume, constant temperature), employing a velocity-Verlet integrator with a time step of Δ*t* = 0.01τ. The LJ time was mapped based on comparing the entanglement time of PDMS in simulations and experiment,^51^ resulting in 1τ = 13 ps. The temperature was maintained by using a Langevin thermostat with a damping constant of γ = 0.02/Δ*t*.

Periodic microstructures with initially *K* = 10^4^ chains with a molar mass *M* that is a multiple (*N* + 1)*m*, closest to the molar mass *M*_*n*_ experimentally measured by Sharaf et al.,^43^ were packed in cubic boxes together with the corresponding number of cross-linker beads *N*_cl_ = 2*rK*/*f* = 5000*r*. Here, *N* is the number of bonds per chain. The polymer networks were then cross-linked using a collision-diffusion MD approach^52^ until the desired target soluble weight fraction *W*_sol_, as measured by Sharaf et al., was reached. In the MD polymerization procedure, equilibrated systems consisting of melt chains of length *N* – 2 as well as *N*_cl_ tetrafunctional cross-linker beads were end-linked by introducing FENE bonds between any unreacted pair of chain-end and cross-linker sites that were separated by less than 1.1σ; the extent of reaction *p* was straightforwardly calculated using the number of the formed bonds during the process. Real polymer chains that cannot pass through each other and themselves were used for polymerization, while the developing networks were on-the-fly processed to calculate *W*_sol_ by using only information about network connectivity, i.e., by assuming phantom chains.^15,16,52^ To determine the weight fraction of the soluble material *W*_sol_ in a microstructure and terminate the polymerization, the fraction of the chains and cross-linkers that were not connected to the infinite network was computed at each step. This allowed to generate representative computer microstructures with reaction extents up to and above *p* = 0.98.^16^ After cross-linking, the soluble part was removed and the size of the simulation box was adjusted to keep the density at 0.85σ^–3^. The extracted network was equilibrated, and stress relaxation MD simulations were performed to determine the shear modulus.

To obtain MD microstructures that mimic the experimentally realized networks, *W*_sol_ was used as the stopping condition in the simulated polymerization process, as described above. Figure 1a shows the exact correspondence of the soluble fraction of network chains between experiment and MD except for two networks that already reached a complete MD polymerization (*p =* 1) for some higher *W*_sol_ values; this means that there are no reactive pairs left to further reduce *W*_sol_ in the MD simulations. These two networks are represented by the two points with *p*_MMT_ = *p*_MD_ = 1 in Figure 1b. This indicates a weakness in relating *p* to *W*_sol_ during the polymerization process in the simulations, which is also observed in the rest of the samples when the estimated extents of reaction by MD and calculated extents of reaction by MMT are compared (Figure 1b). It is apparent that the *p* values obtained from MD are higher than those obtained from MMT, which are increasing with decreasing *r*. This can be attributed to the fact that in calculating the soluble fraction numerically, *W*_sol_ is determined by considering phantom chains, i.e., chains that can cross each other and themselves. As such, entangled loop structures that are in reality trapped in the network by catenated loops are nonetheless counted as soluble material when assuming phantom chains, thus leading to an increased value of *p*_MD_ when using such phantom *W*_sol_ as a stopping criterion in MD polymerization, see Figure 1b. This in turn leads to higher values of the shear modulus when estimated from MD. Therefore, this highlights an inherent weakness in computing *W*_sol_ and the need for caution. This also raises the need for utilizing the extent of reaction, as obtained from MD, as input for MMT instead of *W*_sol_ to make a meaningful comparison between the MMT and MD predictions of the shear modulus. We will label these calculations with a subscript “MD-MMT”.

To extract the shear modulus of the MD networks, three pairs of opposite simple shear strains with a magnitude of γ = ±0.2 are applied; such a relatively high shear strain was chosen in order to reduce the relative fluctuations of *G*(*t*) as compared to its plateau values.^16^ It is known that for simple shear strain used in our work, both Mooney’s and Rivlin’s formulations for general large strain simply reduce to Hooke’s law of linear elasticity, as discussed in Chapter 10 of Treloar’s textbook.^5^ This results in six stress relaxation moduli *G*(*t*) for every system. To obtain the equilibrium shear modulus, the plateau value is extracted by averaging the second half of *G*(*t*). The simulation time is at least an order of magnitude longer than the entanglement time of PDMS (0.11 μs),^44^ and it largely increases upon increasing *M*_n_ and *W*_sol_. The stress relaxation simulations are carried out until no significant decrease in mean *G*(*t*) is observed on a log-time scale. The shear modulus and its error for every system are obtained by taking an average and variance over all six directions. Further details of the approach for computing the shear modulus with examples of input LAMMPS and postprocessing MATLAB scripts are presented in the Supporting Information. The stress relaxation MD simulations took about 100 core-years CPU time on the ETH Zürich Euler cluster. Table 1 provides a summary of the generated computer models and characterization of their network defects.

## Results and Discussion

**Figure 2:**
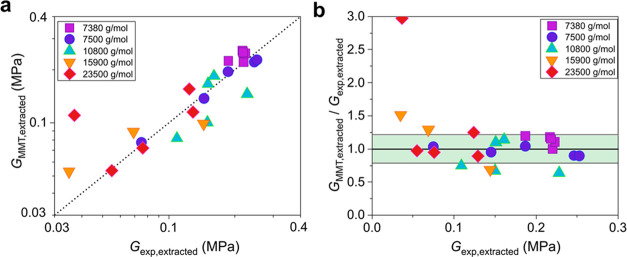
MMT prediction for the experimental data of Sharaf et al.^43^ The relatively large scatter is due to unavoidable experimental inaccuracies in the stoichiometric imbalance, possible side reactions, somewhat different preparation and measurement conditions, etc. (a) The shear modulus calculated from eq 1 assuming *G*_e_(1) = 0.207 MPa is compared with the corresponding measured equilibrium shear modulus. The dotted line is *y* = *x*, and it serves as a guide to the eye. (b) The ratio of the calculated to the measured shear modulus versus the measured value. The shaded green area corresponds to 2σ, where σ is the standard deviation of the ratio of the calculated to the measured shear modulus computed for all of the data points excluding the upper point representing the network prepared with long PDMS chains of *M*_n_ = 23 500 g/mol and *r* = 0.85, which is closer to the gel point so the largest species in the sol may entangle with the network and get stuck in the gel upon extraction, which is treated as an outlier. The black line represents the mean value, while the half-band corresponds to the standard deviation (σ = 0.211) of the statistical population. The mean value of the ratios is 1.014, and the standard error of the mean is SE = 0.046. The subscript “extracted” implies that the scaling (from the swelled to the deswelled state) has been taken into consideration, even though it practically does not affect the results.

Figure 2 shows that the predictions of the shear modulus from the MMT theory are in overall good agreement with the measured moduli of real end-linked networks when *r*, *W*_sol_, and *G*_e_(1) are known. Among these three parameters, *r* and *W*_sol_, which comprise the necessary information about the topological fingerprint of a network, are measurable quantities (provided by Sharaf et al.^43^), while typically for well-developed networks, *G*_e_(1) is assumed to be the plateau modulus of the corresponding high-molar-mass linear polymer melt.^14,53,54^ The plateau modulus of high-molecular-weight PDMS melts, which corresponds to the molecular weight of the chains between entanglements in analogy to polymer networks, is 0.20 MPa.^55^ A least-squares fitting to all 23 examined networks assuming a 95% confidence interval^56^ revealed that the optimum value of *G*_e_(1) is indeed very close to the plateau modulus, *G*_e_(1) = 0.207 ± 0.031 MPa.

The general agreement between the experimentally measured shear modulus of the examined polymer networks and the predicted values using eq 1 of MMT is already quite apparent in Figure 2a, but it becomes clearer in Figure 2b. The polymer network with the lowest modulus was discarded from the statistical population because of its very large deviation from the mean compared to the rest of the values, as is typically done in error analysis. This statistical analysis demonstrates that the deviation of the predicted shear modulus from the measured modulus can be practically attributed only to the random error of repeatability of the measurements since the mean value of the ratios is only about 1.5% higher than unity.

The reason behind the scattering of the values in Figure 2 is related, therefore, to the inaccuracies that are implicitly present when experiments are performed. The stoichiometric imbalance, *r*, of the two participating active groups in the cross-linking reaction is calculated from the amount of the precursor PDMS chains and the amount of the functional silane cross-linking agent as measured by weighing. Apart from the precision of the instrument, impurities present in the two commercial compounds and the molecular weight distribution of the polymer chains make the estimation of *r* inexact. Sample preparation steps and conditions such as mixing rate and curing temperature can also contribute to the scattering. Imperfect mixing may enhance polymer network inhomogeneities, which are large-scale molecular features that influence the properties of the polymer networks.^57^ Slow addition of the cross-linking agents to the precursor chains alters the network formation kinetics and reduces the presence of primary loops resulting in a higher elastic modulus compared to the conventional mixing.^57,58^ Furthermore, the temperature at which the cross-linking reaction takes place plays an important role in the properties of the polymer networks by initiating side reactions or affecting the reaction kinetics. At around 30 °C, redistribution of the cross-linking agent may occur, which increases its functionality, while at higher temperatures the end-groups of the precursor polymer chains can react with their backbone.^42,59^ Thus, the two side reactions may change the stoichiometric ratio between the participating functional groups and in turn the mechanical properties of the resulting networks.^53,54^ It is also important to note that other side reactions may occur in the presence of oxygen and humidity.^59^ Further, an increase in the curing temperature has a direct effect on the reaction kinetics and leads to denser polymer networks with higher shear modulus.^60,61^ Finally, the state of matter of the polymer networks after curing may complicate their handling. Soft polymer networks with low modulus of elasticity and high fraction of soluble material are particularly difficult to handle, which might introduce additional error in the measurement of the modulus and the extraction of the soluble material.^34^ For instance, an underestimation of *W*_sol_ leads to an overprediction of the modulus. This might well be the reason for the high deviation from unity of the ratio *G*_MMT,extracted_/*G*_exp,extracted_ of the network with the lowest shear modulus (the outlier in Figure 2b).

All of these inaccuracies that may happen before, during, and after the curing of model end-linked networks introduce accumulated errors and result in scattered values. The scattered values of the modulus of elasticity have misled theoreticians over the years into establishing an accurate theory that can describe small-strain rubber elasticity based on the underlying topological elements that are involved; the most indicative studies are the work of Patel et al.^42^ and the work of Sharaf and Mark.^54^ Of course, another natural explanation for the misconception of the experimental data lies in the imperfection of the presumably perfect model networks that in fact are not defect-free. Real networks incorporate free and dangling chains and structures because of the incomplete cross-linking as well as low-order loops formed by intramolecular reactions and intermolecular trapped entanglements. However, not all of these are considered in most of the classical theories of rubber elasticity on the basis of statistical thermodynamics as well as in MMT. In this regard, the scattering between the experimentally determined shear modulus and the predicted modulus from theory (Figure 2) is fully justifiable and may be considered as the best-case scenario in comparing theory and experiments when many different networks are involved.^42,43^

The general agreement between experiments and MMT is surprising, though. This theory, in contrast with the Gaussian statistical theories, is a simplified method for deriving average properties of nonlinear polymers utilizing the recursive nature of the stochastic branching theory paired with an elementary law of conditional probability. Typically, the stochastic theory is used for the description of polymers in the pre-gel regime. However, Miller and Macosko extended the description to the post-gel regime as well.^30,31^ By virtue of its straightforward analytical treatment, the theory’s underlying assumptions are oversimplified. In reality, steric hindrance prevents equal reactivity of the functional groups of the cross-linking agents. In addition, it has been proven experimentally and computationally that intramolecular reactions occur during the network formation.^62−64^ However, the branching theory of Miller and Macosko, as all of the classical theories based on the theory of Flory and Stockmayer for the gelation of ideal networks,^14,65,66^ does not take any of the above observations into account. Therefore, one should rather have expected the failure of the theory instead, which is evidently not true. Despite the oversimplified underlying assumptions and the lack of a detailed molecular description, it appears that the MMT can nevertheless accurately describe the equilibrium shear modulus of real model networks. It may be concluded, therefore, that the probabilistic incorporation of the degree of completion, of the dangling chains and segments, and of the trapped entanglements into the topology of a tree-like network is sufficient to predict the shear modulus of rubber-like materials, with a more detailed topological description not necessarily needed.

**Figure 3:**
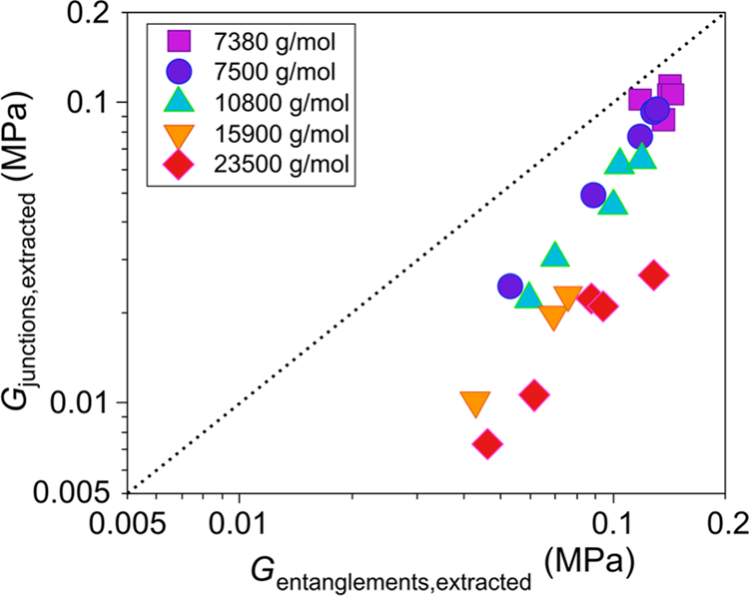
Predictions of MMT decomposed into two contributions, those of the junctions, *G*_junctions,extracted_, and entanglements, *G*_entanglements,extracted_ ≡ *G*_MMT,extracted_ – *G*_junctions,extracted_, for the shear modulus of all of the examined networks of Sharaf et al.,^43^ see eqs 1, 3, and 4. The dotted line is *y* = *x*, and it serves as a guide to the eye.

By decomposing the expression of the shear modulus of eq 1 in the two terms, the junctions and the entanglements contribution, one can plot separately the entanglement and junction contribution to elasticity (i.e., the elastic contribution coming solely from the effective strands of the network without taking into account its trapped entanglements). Figure 3 shows that the contribution from the effective strands is always lower than the contribution from the entanglements, indicating that the contribution from the trapped entanglements is necessary to provide a good estimate of the modulus of the examined networks.

As expected, with longer precursor chains the relative entanglement contribution becomes larger since the junction contribution of the network decreases with increasing precursor chain length; for a given *M*_n_, the higher the value of *r*, the higher the entanglements contribution. However, decomposing *G*_MMT_ into the junction and the entanglement contributions, apart from improving our understanding, also highlights an interesting phenomenon. Networks with short precursor chains appear to have a large contribution from trapped entanglements even below *M*_e_ (≈12 000 g/mol for PDMS), which has also been confirmed by MD simulations for stoichiometric end-linked networks,^16^ as well as for the networks examined in this work (see below), and also for diamond networks.^19^

The macroscopic rheological properties of a melt of short chains, which constitutes the starting point before the onset of cross-linking, are not supposed to be influenced by the entanglements in the first place. However, this does not exclude the possibility of the presence of entanglements. On the contrary, the entanglements are there, but since they are not trapped, they cease to manifest during the measurements. In other words, the entanglements between short chains do not endure the imposed deformation and are easily released, thereby not influencing the rheological properties of their melt. This, on the other hand, may not take place in the course of the cross-linking process, and as a result, the entanglements remain inevitably trapped within the network skeleton thus significantly contributing to its elastic modulus. This phenomenon, which has also been supported by MD simulations,^16^ is rather easily conceptualized, but the critical molecular weight of the precursor chains where the contribution of the entanglements to the modulus becomes dominant over the contribution of the junctions is certainly not intuitive. However, using the equations of MMT, one can approximately find the critical molecular weight of the precursor chains at which the modulus becomes entanglement-dominated. Note that eq 1 states that while the junction contribution to the modulus depends on *M*_n_, the entanglement contribution is independent of *M*_n_.

**Figure 4:**
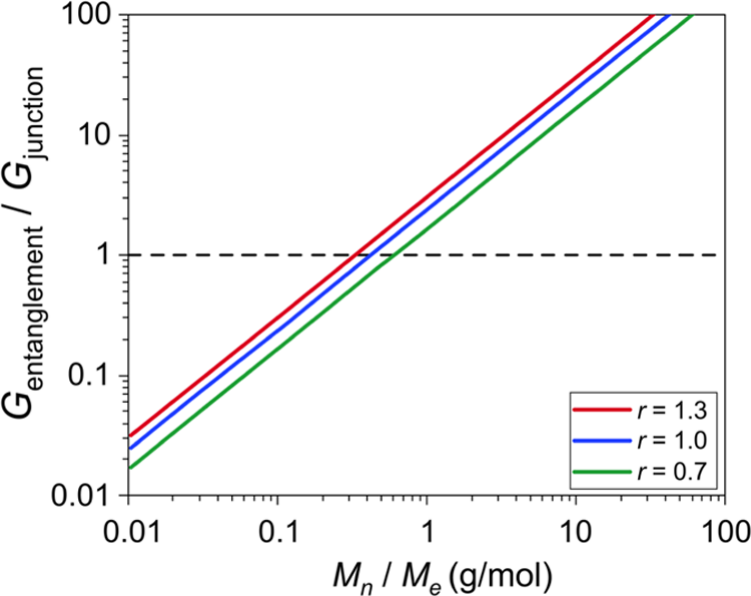
Predictions of MMT [eq 1] for the ratio of the entanglement and junction contributions to the modulus for various values of *M*_n_ and three values of the stoichiometric imbalance *r*, assuming *p* = 0.9. The dashed line serves as a guide to the eye and represents the parity of the entanglement and the junction contribution to the modulus of the end-linked networks. The entanglement molar mass of PDMS is *M*_e_ = 12 000 g/mol.

Figure 4 shows the ratio of the entanglement contribution to the junction contribution, *G*_entanglement_/*G*_junction_, as a function of the molecular weight of the PDMS precursor chains, calculated using three values of *r* and assuming *p* = 0.9. It is seen that for the stoichiometric end-linked networks (*r* = 1) and for *M*_n_ below 5000 g/mol the contribution of the network junctions dominates, while for *M*_*n*_ above 5000 g/mol, the entanglement contribution dominates; since the entanglement molecular weight of PDMS is 12 000 g/mol, this corresponds to about only 42% of *M*_e_. This critical molecular weight shifts toward larger values when *r* < 1 and toward lower values when *r* > 1. For *r* = 0.7, the entanglement contribution becomes dominant at about 7500 g/mol (62.5% of *M*_e_), while for *r* = 1.3, it becomes dominant at about 4000 g/mol (one-third of *M*_e_). In addition to the dependence on the stoichiometric ratio *r*, the behavior depends on the extent of reaction *p* when *r* ≠ 1. It can be easily shown that when *r* < 1 the critical molecular weight at which the entanglement part dominates over the junction part shifts toward larger values with decreasing *p*, while it remains unaltered with increasing *p*. On the other hand, when *r* > 1, this critical molecular weight remains unaltered with decreasing *p*, while it decreases with increasing *p*. Nevertheless, even for *r* = 0.7 and realistically low extents of reaction below *p* = 0.9, the critical molecular weight above which the contribution of entanglements to the shear modulus dominates over the contribution of the junctions is lower than the known entanglement molecular weight of PDMS, *M*_e_ ≈ 12 000 g/mol. Therefore, one may argue that the modulus of all practically relevant end-linked PDMS networks is, in fact, entanglement-dominated.

**Figure 5:**
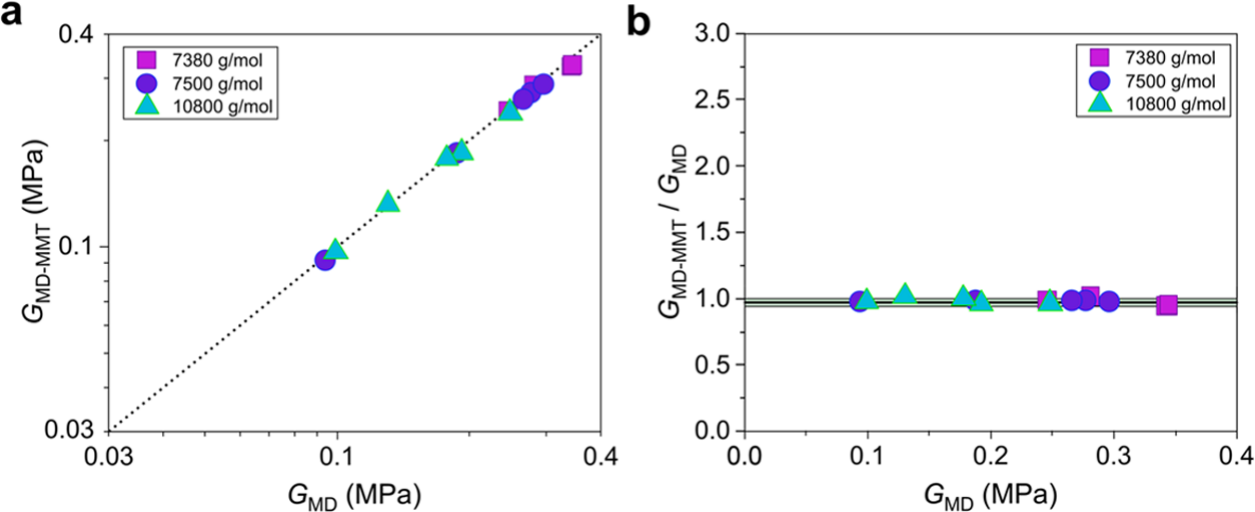
MMT and MD predictions of the experimental data of Sharaf et al.^43^ (a) MMT shear modulus as calculated from eq 1 using the *p* values directly obtained from the MD microstructures (see Figure 1b) and assuming *G*_e_(1) = 0.252 MPa as compared with the corresponding MD predictions of the equilibrium shear modulus for the tetrafunctional end-linked networks with precursor PDMS chains of a molecular weight lower than *M*_e_. The dotted line is *y* = *x*, and it serves as a guide to the eye. (b) Ratio of the shear modulus calculated from MMT to the modulus estimated from MD versus the estimated value. The standard deviation of the ratio of the calculated to the estimated shear modulus computed for all of the data points is about 2.6%. The black horizontal line at 0.97 represents the mean value.

To strengthen our claims and to validate MMT for nonstoichiometric end-linked networks, molecular dynamics simulations were performed to mimic the examined networks built up with chains of molecular weight below the entanglement molecular weight, *M*_e_, of PDMS melts, which are numerically tractable today. Similar graphs as those shown in Figure 2 are presented in Figure 5, but this time the comparison is made between the MD predictions using the experimentally determined *W*_sol_ values^43^ and the MMT predictions using the calculated final extents of reaction from the MD microstructures. This is done to circumvent the mismatch between *W*_sol_ and *p* pairs of the two methods (see Figure 1). That is, by using the same *p*, instead of *W*_sol_, values, the comparison becomes more direct since the weakness of numerically estimating *W*_sol_ with assumed phantom strands has been identified. As demonstrated by Figure 5a,5b, the agreement is almost perfect, i.e., within just 10%, in accordance with our previous observation for stoichiometric networks.^16^ The mean value of the ratios is only about 3% lower than unity, while SE = 0.007 and σ = 0.026. Note that a least-squares fitting to the examined networks assuming a 95% confidence interval revealed that the optimum value of *G*_e_(1) is in this case *G*_e_(1) = 0.252 ± 0.007 MPa, which is indeed very close to 5*G*_N_^0^/4 as recommended by the tube model for the entanglement modulus of fully polymerized stoichiometric networks, as discussed in the paragraph after eq 1.

**Figure 6:**
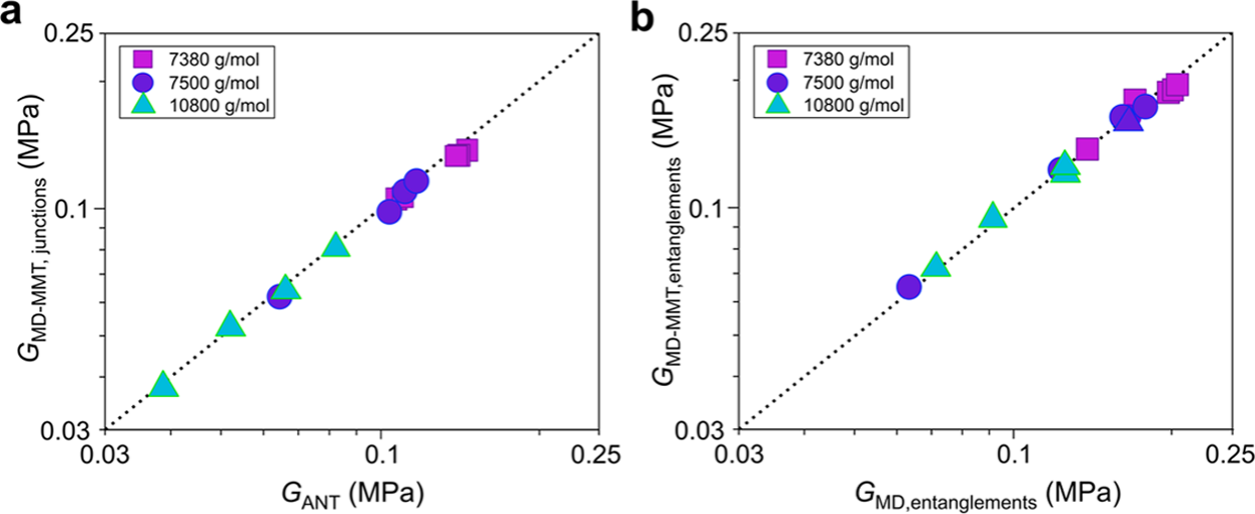
Comparison between (a) the phantom ANT prediction from MD and the junction contribution to elasticity from MMT, and (b) the entanglement contribution to the modulus as estimated from MD and calculated from MMT. The dotted line is *y* = *x*, and it serves as a guide to the eye.

Again in accordance with our previous observation for stoichiometric networks,^16^ by decomposing the junctions’ and entanglements’ contributions to the modulus, it is shown that the former coincides with ANT as calculated from MD and the latter coincides with the difference between the MD estimate for the modulus and the corresponding ANT estimate, i.e., the entanglement contribution obtained from the MD simulations (Figure 6). This is yet more evidence on the accuracy and validity of MMT to predict the equilibrium shear modulus. In addition, Figure 6 shows the large contribution of trapped entanglements to the modulus of the elastomers prepared with chains having a molecular weight lower than *M*_e_. Overall, as expected, the relative contribution of entanglements to the modulus increases with the length of the network chains. However, the significant contribution of entanglements to the modulus of networks with shorter chains is also obvious.

**Figure 7:**
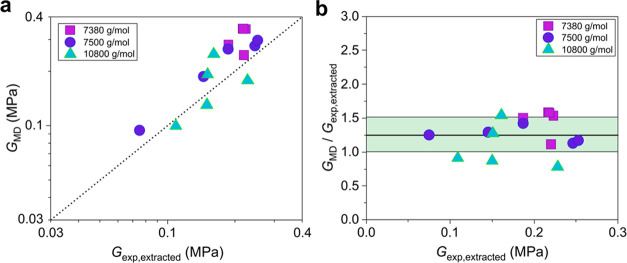
MD predictions of the experimental data of Sharaf et al.^43^ (a) Equilibrium shear modulus computed from the MD simulations using the measured *W*_sol_ values as compared with the corresponding experimental results of the modulus for the networks with precursor PDMS chains of molecular weight lower than *M*_e_. (b) Ratio of the estimated from MD to the experimentally measured shear modulus versus the experimentally measured value. The standard deviation of the ratio of the estimated to the measured small-strain shear modulus is about 26%.

Figure 7 provides a comparison between the MD estimates and the experimentally measured values of the shear modulus. Because an MD microstructure of a network corresponds to a higher extent of reaction *p* than that from MMT for the same *W*_sol_, MD now overpredicts the shear modulus of all of the examined networks (Figure 7a). The mean value of the ratios *G*_MD_/*G*_exp,extracted_ is 1.264 and the standard error of the mean is SE = 0.069, with a standard deviation of σ = 0.258 of the statistical population of the measurements. While, therefore, the statistical error is merely slightly larger than that between the moduli calculated from MMT and the moduli measured experimentally (Figure 2), the mean value of the ratios now is about 26.5% larger than unity, i.e., more than 17 times higher than that in Figure 2. This is attributed to the higher extents of reaction reached in MD networks and identifies the shortcomings of the numerical calculation of the fraction of the soluble material of the elastomer assuming phantom strands. As discussed above, when calculating *W*_sol_ at each step of the MD polymerization, the intermolecular interactions are neglected even though the MD polymerization itself takes into account the nonbonded interactions. This in turn may lead to the removal of entangled loop structures that are supposed to be trapped in the formed network but are nevertheless counted as soluble material, thereby leading to increased *p* values, and therefore, the obtained shear modulus from the MD simulations is increased. On the other hand, MMT assumes a simplified tree-like representation for the networks, where no intramolecular bond formation is allowed. However, while it is true that both MD and MMT inherently involve oversimplifications, incorporating the topological fingerprint of a network in a probabilistic manner and letting *G*_e_(1) as a free parameter in the range between *G*_N_^0^ and 5*G*_N_^0^/4 compensates for this weak point in MMT (see Figure 2). Note that there may also exist additional computational sources of error, such as uncertainties in the mapping factors between the LJ and SI units and also slow stress relaxation after deformation that would tend to produce overestimations of the modulus.

## Conclusions and Perspectives

We have shown that the predictions of the nonlinear polymerization theory of Macosko and Miller combined with the additive contribution of entanglements are in overall good agreement with the experimental values of the elastic modulus of various end-linked PDMS networks. In predicting the modulus from MMT, the entanglement modulus of fully polymerized stoichiometric networks, *G*_e_(1), is kept as a fitting parameter but is within the expected range [*G*_N_^0^, 5*G*_N_^0^/4]. While MMT oversimplifies the actual network structure by prohibiting the formation of intramolecular loops, this apparently does not influence practical predictions of the shear modulus of end-linked polymer networks formed from bulk, where the fractions of the loops are small, see Table 1 and refs (16,62−64). It becomes apparent, therefore, that for a polymer network formed from bulk, it is sufficient to account for the topological details of a reference tree-like network, i.e., for its degree of completion, junction functionalities, and trapped entanglements, in order to practically predict the equilibrium shear modulus. The validity of the theory was also demonstrated by MD simulations that mimic the experimentally realized networks. A shortcoming is identified in the computation procedure used to estimate the fraction of the soluble material, which is attributed to the neglect of the intermolecular interactions. This results in a higher extent of reaction and, in turn, an overestimation of the equilibrium shear modulus from MD simulations. This shortcoming suggests that caution is needed when using phantom methods to estimate *W*_sol_, and that further efforts are required to estimate *W*_sol_ more accurately in future computational works. Finally, the large contribution of trapped entanglements to the modulus of networks prepared with both short (with a molecular weight lower than *M*_e_) and long precursor chains is presented here, suggesting that the entanglements not only significantly contribute to rubber-like elasticity but also that the elastic modulus of commonly used end-linked PDMS networks is in fact entanglement-dominated.

## Appendix

For the simple uniaxial extension type of deformation, the equation of state under the assumption of affine deformation (ANM) is given by
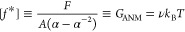
11where [f*] represents the reduced stress, *F* is the tensile force, α is the deformation factor or otherwise the extension ratio, *A* is the cross-sectional area of the sample in the undeformed state, ν is the number density of the network strands (i.e., the chain segments between two consecutive cross-linkers along the chain), *k*_B_ is the Boltzmann constant, and *T* is the temperature. In this form, eq 11 represents the shear modulus of the network, *G*_ANM_.^14^

Through the relaxation in the movement of the junctions, the shear modulus of an ideal network as predicted from PNM acquires the same form as ANM and differs only by the front factor
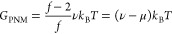
12where *f* is the functionality of the network cross-linkers and μ is the number density of the cross-linkers.^14^

The expression for the modulus as obtained from ANT is given again by the same form as eqs 11 and 12, and it is different only by the front factor
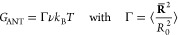
13where Γ is the topological factor which describes the size of the chains in the undeformed state, **R̅** is the mean end-to-end vector of a strand, and *R*_0_ is the mean end-to-end distance of the corresponding chain in a melt state. The brackets correspond to the ensemble average over the strands.^16,67^

In eq 1 of MMT, the following complementing expressions are used
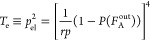
14
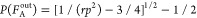
15
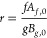
16
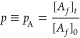
17
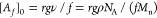
18where *T*_e_ represents the trapping factor [eq 14], *P*(*F*_A_^out^) is the probability of the event that looking out from a reactive site of a network junction chosen at random leads to a finite chain rather than to the infinite network [eq 15, which is stated for a specific case of *f* = 4 and *g* = 2 studied in this work], *r* is the initial ratio of the number of the two participating functional sites with *f* being the functionality of the cross-linker *A*_*f*_ and *g* the functionality of the chains *B*_*g*_ whose number-averaged molecular weight is *M*_*n*_ [eq 16], *p* is the extent of reaction after some time *t* elapsed (i.e., after a fraction of sites have reacted) [eq 17], and [A_*f*_]_0_ is the initial number density of cross-linkers *A*_*f*_. *p*_el_ in eq 14 is the probability that a randomly chosen network strand is elastically effective, i.e., that looking out from each of its two ends leads to the infinite network. ρ and *N*_A_ are the density and the Avogadro number, respectively.

The effective number density of network chains calculated from the recursive theory of Miller and Macosko is given by
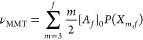
19and the effective number density of network junctions μ_MMT_ by
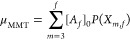
20where
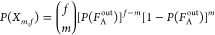
21is the probability that a randomly chosen cross-link *A*_*f*_ is an effective network junction of degree *m*, and the front factor is the binomial coefficient.

## Supporting Information Available

Computational details of the approach for computing the shear modulus, examples of input LAMMPS, and postprocessing MATLAB scripts (PDF)
