# Supplementary material for: A Comparison between Predictions of the Miller–Macosko Theory, Estimates from Molecular Dynamics Simulations, and Long-Standing Experimental Data of the Shear Modulus of End-Linked Polymer Networks
Source: Macromolecules. 2024 Apr 17;57(9):4273–84. doi: 10.1021/acs.macromol.3c02544 (PMC11100001; doi:10.1021/acs.macromol.3c02544)
Supplement: Supplementary file 1 — ma3c02544_si_001.pdf [file ma3c02544_si_001.pdf]

Supporting Information

A Comparison Between Predictions of the Miller-Macosko Theory, Estimates from Molecular Dynamics Simulations and Long-Standing Experimental Data of the Shear Modulus of End-Linked Polymer Networks

Ioanna Ch. Tsimouri, Fabian Schwarz*, Tim Bernhard, Andrei A. Gusev*

Department of Materials, ETH Zürich, CH-8093 Zürich, Switzerland

*E-mails: fabian.schwarz@mat.ethz.ch; gusev@mat.ethz.ch

Details of the approach for computing the shear modulus

The sections below further clarify and detail the steps required to compute the shear modulus of periodic computer models of polymer networks, as developed, implemented and used in our previous works Gusev and Schwarz, *Macromolecules* **55**, 8372 (2022) and ibid., **52**, 9445 (2019), which are cited as refs. [16] and [17] in the main text, respectively. Examples of related LAMMPS and MATLAB scripts are also provided.

Deformation and subsequent relaxation. Each computer network model is deformed by applying a simple shear deformation with a rate of ±0.04 for 5τ, leading to a total shear strain of ±0.2. In the "in.shear" script below, a negative shear strain is applied in the *xy*-plane. Separate simulations are carried out for negative and positive shear deformations in the *xy*-, *xz*- and *yz*-planes. After the deformation, the network model is relaxed and the time evolution

of the respective shear-stress component is recorded to the output file. For every network, this results in six stress-relaxation curves.

In the stress-relaxation runs, a timestep of 0.01τ is typically used. To make sure that the simulations capture the fast deformation dynamics as well as the initial phase of the stress-relaxation dynamics, the timestep is lowered to 0.001τ for the deformation runs as well as for the first two million timesteps of the relaxation runs. For each Cartesian plane, the stress-relaxation data of a deformed network are spread over several files obtained in separate consecutive runs. From the second run onward, the network is simply further relaxed without imposing any additional deformation, see the in.shear2 script as an example.

The stress-relaxation runs commonly consist of 60 million timesteps, except for the $N = 46$, $r = 1.226$ networks where the runs consist of 80 million timesteps and the $N = 46$, $r = 1.420$ and $N = 66$, $r = 1.036$ and $N = 66$, $r = 1.200$ networks where the runs are extended to 200 million timesteps, where N is the number of FENE bonds per network strand, see Table 1 of the main text. These networks require longer relaxation runs because of their larger dangling structures, which are mainly due to the lower degrees of polymerization and longer precursor chains.

Post-processing. For every computer model, six stress-relaxation curves are extracted from the LAMMPS log files using the readLogFastINIT.m and readLogFast.m MATLAB scripts. Subsequently, the six curves are averaged to obtain the stress-relaxation modulus $G(t)$ and the equilibrium shear modulus is estimated as an average over the second half of $G(t)$ using the post_proc.m script. The variance is estimated using the moduli obtained in three different Cartesian planes. A linear fit to the second half of $G(t)$ is used to assess whether or not the stress-relaxation runs have converged.

LAMMPS input files for the deformation and subsequent relaxation (in.shear and in.shear2)

```
#LAMMPS input file: Shear deformation and subsequent relaxation to calculate
the shear stress
package             omp 1

units               lj
atom_style          angle
special_bonds       fene

bond_style          fene

bond_style          fene
pair_style          lj/cut 1.12246
pair_modify         shift yes
angle_style         fourier/simple/omp

read_data           M9672N45_equil_50M.out

change_box          all triclinic

neighbor            0.4 bin
neigh_modify        every 1 delay 1


fix             1 all nve
fix             2 all langevin 1.0 1.0 0.5 904297

thermo          10
timestep        0.001

#deform the simulation box at a constant rate
fix             3 all deform 1 xy erate -0.04 remap v

#compute shear stress
variable            sigt equal -pxy

thermo_style        custom step press v_sigt

comm_modify         mode single cutoff 1.9 vel no

run    5000
unfix        3
run    2000000

thermo              1
timestep            0.01
reset_timestep      200000

run     9800000

#write out the deformed configuration
write_data      deformed_M9672N45_10M.out
```

```
#LAMMPS input file: relaxation to calculate the shear stress
package         omp 1

units               lj
atom_style          angle
special_bonds       fene

bond_style          fene
pair_style          lj/cut 1.12246
pair_modify         shift yes
angle_style         fourier/simple/omp

read_data           deformed_M9672N45_10M.out

neighbor            0.4 bin
neigh_modify        every 1 delay 1

fix                         1 all nve
fix                         2 all langevin 1.0 1.0 0.5 904297

thermo              1
timestep            0.01

#compute shear stress
variable            sigt equal -pxy

thermo_style        custom step press v_sigt

comm_modify         mode single cutoff 1.9 vel no

run    10000000

#write out the deformed configuration
write_data      deformed_M9672N45_20M.out
```

MATLAB scripts to extract the stress-relaxation and equilibrium shear moduli from the LAMMPS readLogFastINIT.m, readLogFast.m and post_proc.m logfiles.

```
function [data_out] = readLogFastINIT(filename,N)

    try
        fid = fopen(filename,'r');
    catch
        error('Data file not found!');
    end

    l_cur= fgetl(fid);

    while length(l_cur) < 4 || ( length(l_cur) >= 4 && ~strcmp(l_cur(1:4),'Step'))
        l_cur= fgetl(fid);
    end

    l_cur=fgetl(fid);

    while length(l_cur) < 4 || ( length(l_cur) >= 4 && ~strcmp(l_cur(1:4),'Step'))
        l_cur= fgetl(fid);
    end


    data1 = textscan(fid,'%f %f %f',200000);
    size(data1{3}(:),1)


    l_cur=fgetl(fid);
    while length(l_cur) < 4 || ( length(l_cur) >= 4 && ~strcmp(l_cur(1:4),'Step'))
        l_cur= fgetl(fid);
    end

    data2 = textscan(fid,'%f %f %f',800000);

    l_cur=fgetl(fid);
    while length(l_cur) < 4 || ( length(l_cur) >= 4 && ~strcmp(l_cur(1:4),'Step'))
        l_cur= fgetl(fid);
    end

    data3 = textscan(fid,'%f %f %f',N);

    data_out(:,1) = [data1{1}; data2{1}; data3{1}];
    data_out(:,2) = [data1{3}; data2{3}; data3{3}];
```

```
function [data_out] = readLogFast(filename,N)

    try
        fid = fopen(filename,'r');
    catch
        error('Data file not found!');
    end

    l_cur= fgetl(fid);

    while length(l_cur) < 4 || ( length(l_cur) >= 4 && ~strcmp(l_cur(1:4),'Step'))
        l_cur= fgetl(fid);
    end

    data = textscan(fid,'%f %f %f',N);

    data_out(:,1) = data{1};
    data_out(:,2) = data{3};
```

```
% extract shear modulus from log files fast

data1_1 = readLogFastINIT('xy/pos/log.lammps10M',9000001);
data1_2 = readLogFast('xy/pos/log.lammps20M',10000001);
data1_3 = readLogFast('xy/pos/log.lammps30M',10000000);
data1_4 = readLogFast('xy/pos/log.lammps40M',10000000);
data1_5 = readLogFast('xy/pos/log.lammps50M',10000000);
data1_6 = readLogFast('xy/pos/log.lammps60M',10000000);

data2_1 = readLogFastINIT('xy/neg/log.lammps10M',9000001);
data2_2 = readLogFast('xy/neg/log.lammps20M',10000001);
data2_3 = readLogFast('xy/neg/log.lammps30M',10000000);
data2_4 = readLogFast('xy/neg/log.lammps40M',10000000);
data2_5 = readLogFast('xy/neg/log.lammps50M',10000000);
data2_6 = readLogFast('xy/neg/log.lammps60M',10000000);

data3_1 = readLogFastINIT('xz/pos/log.lammps10M',9000001);
data3_2 = readLogFast('xz/pos/log.lammps20M',10000001);
data3_3 = readLogFast('xz/pos/log.lammps30M',10000000);
data3_4 = readLogFast('xz/pos/log.lammps40M',10000000);
data3_5 = readLogFast('xz/pos/log.lammps50M',10000000);
data3_6 = readLogFast('xz/pos/log.lammps60M',10000000);

data4_1 = readLogFastINIT('xz/neg/log.lammps10M',9000001);
data4_2 = readLogFast('xz/neg/log.lammps20M',10000001);
data4_3 = readLogFast('xz/neg/log.lammps30M',10000000);
data4_4 = readLogFast('xz/neg/log.lammps40M',10000000);
data4_5 = readLogFast('xz/neg/log.lammps50M',10000000);
data4_6 = readLogFast('xz/neg/log.lammps60M',10000000);

data5_1 = readLogFastINIT('yz/pos/log.lammps10M',9000001);
data5_2 = readLogFast('yz/pos/log.lammps20M',10000001);
data5_3 = readLogFast('yz/pos/log.lammps30M',10000000);
data5_4 = readLogFast('yz/pos/log.lammps40M',10000000);
data5_5 = readLogFast('yz/pos/log.lammps50M',10000000);
data5_6 = readLogFast('yz/pos/log.lammps60M',10000000);
```

```
data6_1 = readLogFastINIT('yz/neg/log.lammps10M',9000001);
data6_2 = readLogFast('yz/neg/log.lammps20M',10000001);
data6_3 = readLogFast('yz/neg/log.lammps30M',10000000);
data6_4 = readLogFast('yz/neg/log.lammps40M',10000000);
data6_5 = readLogFast('yz/neg/log.lammps50M',10000000);
data6_6 = readLogFast('yz/neg/log.lammps60M',10000000);

sigma_xy_pos = [data1_1(:,2); data1_2(2:end,2); data1_3(2:end,2);
data1_4(2:end,2); data1_5(2:end,2); data1_6(2:end,2)];
sigma_xy_neg = [data2_1(:,2); data2_2(2:end,2); data2_3(2:end,2);
data2_4(2:end,2); data2_5(2:end,2); data2_6(2:end,2)];
sigma_xz_pos = [data3_1(:,2); data3_2(2:end,2); data3_3(2:end,2);
data3_4(2:end,2); data3_5(2:end,2); data3_6(2:end,2)];
sigma_xz_neg = [data4_1(:,2); data4_2(2:end,2); data4_3(2:end,2);
data4_4(2:end,2); data4_5(2:end,2); data4_6(2:end,2)];
sigma_yz_pos = [data5_1(:,2); data5_2(2:end,2); data5_3(2:end,2);
data5_4(2:end,2); data5_5(2:end,2); data5_6(2:end,2)];
sigma_yz_neg = [data6_1(:,2); data6_2(2:end,2); data6_3(2:end,2);
data6_4(2:end,2); data6_5(2:end,2); data6_6(2:end,2)];

eps = 0.2;
G_data_xy = 0.5*( sigma_xy_pos / eps - sigma_xy_neg / eps );
G_data_xz = 0.5*( sigma_xz_pos / eps - sigma_xz_neg / eps );
G_data_yz = 0.5*( sigma_yz_pos / eps - sigma_yz_neg / eps );
G_data = (G_data_xy + G_data_xz + G_data_yz)/3;

t_data = (0:1:(size(G_data_xy,1)-1))';

is = 30000001;
G_eq_xy = mean(G_data_xy(is:end));
G_eq_xz = mean(G_data_xz(is:end));
G_eq_yz = mean(G_data_yz(is:end));
G_eq = mean(G_data(is:end));

G_eq_err = sqrt( power(G_eq_xy-G_eq,2) + power(G_eq_xz-G_eq,2) + power(G_eq_yz-
G_eq,2) ) / sqrt(2);

Gp = G_data(is:end);
tp = t_data(is:end); tp2 = tp.*tp;
Np = size(tp,1);
XtX = [Np sum(tp); sum(tp) sum(tp2)];
XtX_inv = inv(XtX);
var_s = XtX_inv(2,2)*var(Gp);

pf = polyfit(tp,Gp,1);
s = pf(1);
T = sqrt(s*s/var_s);

filename = sprintf('G_info_M10000_N45.dat');
fileID = fopen(filename,'w');
fprintf(fileID,'# G_eq | G_eq x | G_eq y | G_eq z | err | s | var_s | T\n');
fprintf(fileID, '%10.8f %10.8f %10.8f %10.8f %10.8f %10.8f %10.8f %10.8f\n',
G_eq, G_eq_xy, G_eq_xz, G_eq_yz,G_eq_err,s,var_s,T);
fclose(fileID);

filename = sprintf('G_data_phi_M10000_N45.dat');
fileID = fopen(filename,'w');
fprintf(fileID,'# t | G(t)\n');
for i=1:size(G_data,1)
    fprintf(fileID, '%d %10.8f\n', t_data(i), G_data(i));
end
fclose(fileID);
```
